# Abortion incidence and safety in Niger in 2021: Findings from a nationally representative cross-sectional survey of reproductive-aged women using direct and indirect measurement approaches

**DOI:** 10.1371/journal.pgph.0002353

**Published:** 2023-10-13

**Authors:** Suzanne O. Bell, Sani Oumarou, Elizabeth A. Larson, Souleymane Alzouma, Caroline Moreau

**Affiliations:** 1 Department of Population, Family and Reproductive Health, Johns Hopkins University Bloomberg School of Public Health, Baltimore, Maryland, United States of America; 2 Institut National de la Statistique, Niamey, Niger; 3 Soins Primaires et Prévention, Centre de Recherche en Epidémiologie et Santé des Populations, U1018, Inserm, Villejuif, France; Pontifical Catholic University of Peru: Pontificia Universidad Catolica del Peru, PERU

## Abstract

Niger is a country in which legal restrictions and a dearth of research has long limited our understanding of the extent and safety of induced abortion. The current study is the first national study of induced abortion in Niger. It uses direct (self-report) and indirect (best friend method) to provide nationally representative estimates of induced abortion incidence and safety and evaluates the performance of the indirect measurement approach. We used cross-sectional, representative survey data on women aged 15–49 in Niger collected between January and May 2022; final sample included 3,696 women. The survey included questions on respondents’ and their closest female friends’ experience with abortion, including methods and sources used. We calculated one-year abortion incidence and the proportion of abortions involving non-recommended methods and/or sources to determine safety separately for respondents and friends, overall and by background characteristics. The fully adjusted one-year friend abortion rate was 6.7 abortions per 1,000 women in 2021, which was substantially higher than the corresponding respondent rate of 0.4 per 1,000 women. Confidence intervals were wide, but friend estimates suggest higher abortion rates among women in their 20s, those with secondary or higher education, and those with no children. Nearly all abortions were unsafe (97% respondents, 100% friends), involving non-recommended methods and/or sources. While abortion numbers were small, unsafe abortion appeared more common among older women, married women, those with children, and those residing in rural areas. Our findings indicate that, despite legal restrictions, some women undergo abortions in Niger at great risk to their physical safety. Ensuring adequate access to quality voluntary family planning services to prevent unintended pregnancy and postabortion care to treat complications is essential to reducing the risk of unsafe abortion in the country.

## Introduction

Induced abortion occurs in all societies regardless of legality, often taking place clandestinely outside the formal healthcare sector, making it difficult to measure and monitor [1,2]. The ability to design evidence-based policies and programs to improve family planning services, postabortion care, and safe abortion to the limit of the law, depends on the capacity to measure the incidence of induced abortion across sociodemographic groups within a specific geography and time period. Induced abortion is also of great public health significance as it remains a leading cause of maternal morbidity and mortality despite the safety of the procedure when done in accordance with clinical guidelines [3]. Unsafe abortion accounts for between 8% and 15% of maternal mortality worldwide, resulting in the deaths of approximately 25,000 to 44,000 women annually [4,5]. Nearly all abortion-related mortality and morbidity occurs in settings with restrictive abortion laws and insufficient access to safe abortion and postabortion care services, where women rely on unsafe means to terminate unwanted pregnancies [6].

Niger is a country in which legal restrictions on induced abortion and a dearth of research on the topic has long limited our understanding of the extent and safety of induced abortion and its impact on the country’s health and demographic indicators. Niger–a Sahelian country of more than 25 million people who reside overwhelmingly in rural areas–has the highest total fertility and desired fertility rates in the world (7.6 and 7.4, respectively) [7], the lowest rate of unintended pregnancy (49 per 1,000 women aged 15–49 in 2015–2019) [8], and among the lowest levels of contraceptive use (11%) [9], thus we would anticipate low levels of abortion given a low demand for fertility limitation regardless of abortion legal restrictions. Induced abortion is legally permitted in Niger only to save the life of the woman or to preserve her health (with no mention of mental or physical health specifically) or in the case of fetal abnormalities [10]. Global models estimate there are approximately 15 induced abortions per 1,000 reproductive aged women annually in Niger, though no country-specific study has sought to measure the incidence or its correlates [2]. While there are no estimates of abortion safety in Niger, estimates for West Africa suggest as much as 85% of abortions in the region are unsafe [11]. Maternal mortality in the country is high at 441 deaths per 100,000 live births, approximately 10% of which is attributable to unsafe abortion based on regional estimates for sub-Saharan Africa [5,12]. One small retrospective study among 151 women who received postabortion care at a referral maternity hospital in the capital, Niamey, following illegal abortions found that 57% experienced either infection or incomplete abortion, and that almost 85% required some form of hospitalization [13]. Furthermore, over 8% of these 151 women died due to complications stemming from unsafe abortion. These findings demand further understanding of the extent, safety, and correlates of abortions occurring in Niger in order to reduce associated maternal morbidity and mortality. However, the legal restrictions and stigma surrounding abortion make it difficult to study.

In recent years, researchers have more frequently implemented social network-based approaches to measure highly sensitive behaviors that are underreported using direct questioning [14–18]. These approaches ask respondents to report on their friend’s behavior, instead of their own, in order to reduce social desirability bias, with variations based on the number of friends included and the friend relationship criteria [15–17,19]. These social network-based approaches make three broad assumptions: first, that the friend surrogate sample is similar to the respondent sample and therefore representative of the population of reproductive-aged women in a given geography (i.e., no selection bias); second, that respondents are aware of the sensitive behaviors of their friends (i.e., no transmission bias); and third, that respondents are more likely to report their friends’ sensitive behavior than their own (i.e., no social desirability bias). Performance of these social network-based methods and the extent of assumption violations have been mixed, although friend abortion estimates are consistently higher than those generated from self-reports [20–27]. Although no such methodology has been implemented in Niger, it has performed well in similar settings such as Burkina Faso, Cote d’Ivoire, Nigeria, and other sub-Saharan African contexts [21–23,27].

The current study is the first national study of induced abortion in Niger. Given the demographic and public health impacts of induced abortion, we sought to provide critical insight to our understanding of the practice of induced abortion in the country. Using population-based survey data of reproductive-aged women, we asked about respondent’s and their closest female friend’s experience with abortion. The study aims to provide national estimates of induced abortion incidence and safety in Niger, overall and by women’s sociodemographic characteristics, and to evaluate the performance of the indirect approach (the best friend approach) used.

## Methods

### Data

Survey data came from the Performance Monitoring for Action (PMA) Niger study, implemented by the Institut National de la Statistique (INS) du Niger (Niger National Statistics Agency) with technical support from the Bill & Melinda Gates Institute for Population and Reproductive Health at the Johns Hopkins Bloomberg School of Public Health [28]. In Niger, PMA conducts a nationally representative panel survey of households and reproductive-aged women that monitors sexual and reproductive health indicators and explores family planning dynamics annually. The Phase 1 (baseline) sample size was determined based on the number of respondents required to estimate the modern contraceptive prevalence within a three-percentage point margin of error at the national level and a five-percentage point margin within urban and rural strata. The survey used a multi-stage cluster sampling design with urban-rural strata. In Phase 1 (baseline), conducted from December 2020 to April 2021, researchers randomly selected 103 geographic clusters from the strata using probability proportional to size, then interviewers listed all households within each cluster. Next, PMA randomly selected 35 households from each cluster and invited all women aged 15–49 in those households to participate in the survey. Interviewers obtained verbal informed consent before conducting the interview. Additional details on PMA survey methodology can be found elsewhere [29]. In total, interviewers completed 3,515 household surveys (98.8% response rate) and 3,666 female surveys (95.4% response rate) in Phase 1.

Data for this study came from Phase 2, which was conducted from January 2022 through May 2022 and included a module on abortion in the female survey. We included all panel women who completed the Phase 2 survey and who still resided in sample clusters (n = 2,831, 83.9% follow-up). PMA randomly selected replacement households equal to the number of households lost to follow-up from within clusters that had more than 10% loss to follow-up to counteract attrition in order to produce nationally representative cross-sectional estimates of indicators. To identify these replacement households, interviewers first updated the cluster sampling frame by listing all households in advance of Phase 2. After panel data collection, interviewers randomly selected the number of households lost to follow-up from the updated cluster sampling frame and invited these households to participate in the study. Including the replacement households, the final Phase 2 sample used for this study was 3,428 households (98.8% response rate) and 3,696 women aged 15–49 (96.3% response rate). We constructed survey-design weights using the inverse of the cluster and household selection probabilities and further adjusted the weights for non-response at the household and individual level within each cluster. Local interviewers conducted the surveys face-to-face, collecting data on respondents’ sociodemographic characteristics, reproductive history, knowledge and use of family planning, as well as their and their closest friend’s experience with abortion. Responses were entered by interviewers via a smartphone application. Interviews were conducted in French, which was programmed into the data collection application, or a local language using verbal translations decided upon during training. The survey received ethical approval from the Institutional Review Board at the Johns Hopkins University Bloomberg School of Public Health (#14590) and Comite National d’Ethique pour la Recherche en Santé (CNERS) in Niger (#078/2021/CNERS).

### Measures

PMA added an abortion module to Phase 2 of the female survey. This module measured the abortion experiences of respondents and their closest female friends via two sets of questions, the first asking about whether they had ever done something to end a pregnancy and the second asking about ever doing something to bring back a late period, and if so, whether the woman took action because she thought she was pregnant as women may regulate their periods for reasons other than fertility management [30,31]. We included all successful attempts to intentionally end a pregnancy or bring back a period when the woman thought she was pregnant in our definition of abortion in this study. PMA asked additional questions on the most recent abortion experience, including the year it occurred and the source(s) and method(s) used.

We grouped information on specific abortion methods and sources into broader categories for analyses. Method categorizations included surgery (no additional details collected on type of surgery), medication abortion pills (misoprostol with or without mifepristone), other pills identified by the respondent (emergency contraception, contraceptive pills, antibiotics or anti-malarial pills), pills that the respondent could not identify, and traditional or “other” methods (herbs, bleach, inserted items, and “other”). Respondents could also indicate “do not know” or provide no response. Abortion sources were grouped into four categories, including public facilities, private facilities, pharmacies, and other non-clinical sources. Public facilities included the following facility types: Maternity Central, Regional Hospital Maternity Center, District Hospital Maternity Center, Health Center, Case de Santé, and Public Mobile Clinics. We categorized Private Hospital or Clinic, Private Mobile Clinics, Nigerien Association for Family Well Being (ANBEF) Center, and Polyclinic or Private Clinic as private facilities. We grouped public and private pharmacies together in one pharmacy category, and all other service delivery points we considered as other non-clinical sources, which included Community-Based Distribution Site, Kiosk Routier, Boutique, religious organizations, community event, friend/parent, walking pharmacy, and “other” sources.

The World Health Organization’s (WHO) defines safe abortions as those that are conducted by trained providers in an appropriate clinical context using recommended methods (i.e., appropriate abortion surgery or misoprostol with or without mifepristone), less safe abortions as those that involve either trained providers or a recommended method, and least safe abortions as those that involve both untrained providers and non-recommended methods [11]. Using data on abortion source(s) and method(s), we categorized our abortion safety indicator based on WHO’s definition [11] with some caveats due to data limitations. Specifically, as respondents could not provide information on provider training, we assumed abortions from any health care facility (the first two categories in the aforementioned abortion sources variable) were conducted by trained providers, while all other sources involved untrained providers. We classified abortion surgeries and medication abortions using misoprostol with or without mifepristone as involving a recommended method, and all other methods (other/unknown pills, injections, and traditional methods) as involving non-recommended methods. Given data constraints we framed these categories as abortions involving a recommended method and source (analogous to safe), non-recommended method or source (less safe), and non-recommended method and source (least safe). We created a second measure of abortion safety to reflect the most recent WHO safe abortion guidelines, which consider self-managed medication abortions using recommended abortion pills to be safe (as opposed to less safe in the previous definition) [32]. We thus recategorized abortions involving medication abortion pills that were not obtained in healthcare facilities as safe in this secondary analysis. We used this reclassification to dichotomize abortion safety into safe (recommended method and source) versus unsafe (non-recommended method and/or source) to examine correlates of abortion safety.

We used the following indicators for respondents and friends to examine correlates of abortion incidence and safety: age (15–19, 20-29-, 30–39, 40–49), highest education level attended (never attended, primary, secondary or higher), marital status (in union (married/cohabiting), not in union), residence (rural, urban), and parity (nulliparous, parous). To assess methodological assumptions, we also examine current contraceptive use (no, yes) and current long-acting reversible contraceptive (LARC) use (no, yes). For respondents only, we also looked at household wealth tertiles, defined based on a score derived from principal components analysis using information on household assets, water, sanitation, and building materials, which is similar to the approach implemented by the Demographic and Health Surveys.

### Analysis

We began our analysis by identifying and adjusting for potential selection and transmission biases using a five-step approach. First, we tested for sociodemographic differences between respondents who had a close female friend and those who had no friends using design-based F-tests. Second, we incorporated the data from respondents who had no friends into the surrogate friend sample, including data on abortion methods and sources. We did so based on the assumption that women with no friends are inherently missing from the friend surrogate sample but would have similar sociodemographic characteristics as respondents with no friends. We refer to these data as the adjusted surrogate sample.

Third, in order to more accurately capture a respondent’s likelihood of having had a recent abortion given known biases in self-reported abortion data [14], we used the adjusted surrogate sample and regressed the sociodemographic characteristics on abortion incidence. We then used the predicted probability of abortion in the last year from this model to generate the likelihood of a respondent with no friends having had an abortion in the previous year, thus omitting the self-reported abortion data from the adjusted surrogate sample.

Fourth, we generated post-stratification weights to further adjust the surrogate sample and align its sociodemographic distribution with the characteristics of the respondent sample, which was designed to be nationally representative of reproductive-aged women. We created these weights by regressing the sociodemographic characteristics of respondents and the adjusted surrogate sample on whether the observation was from the original respondent sample or from the adjusted surrogate sample and taking the inverse predicted probability of being in the adjusted surrogate sample. We then multiplied the post-stratification weights and the PMA survey design weights to produce the final adjusted surrogate sample weights.

The final step was to account for respondent’s incomplete knowledge of their friend’s abortions (transmission bias). To do so, we used results from the question, “Do you think your friend, [respondent provided fake name of friend], knows about this event?”. The transmission bias was then calculated as the inverse proportion of respondents who reported “Yes” or “Maybe” to whether their friend knew about their own abortion experience [24–26]. In using this approach, we assumed that the abortion sharing patterns of respondents with friends is similar to the sharing patterns of friends with respondents (and respondents who did not report their abortion). We then multiplied the adjusted surrogate sample induced abortion incidence estimates by the transmission bias adjustment factor.

After applying these adjustments, we first compared the one-year abortion incidence of the respondents to that of the friends, overall and by sociodemographic factors. To account for potential displacement across years, we included abortions reported to have taken place in 2021 and early 2022 and divided all reported abortions by the average number of person-years (1.19) to compute the one-year abortion incidence rates for both respondents and friends and multiplied the rates by 1,000. We then examined the distribution of abortion methods, sources, and safety for respondents and friends and explored the sociodemographic characteristics associated with unsafe abortions for both populations.

All analyses were conducted in Stata version 17 [33], used survey design weights to account for the complex sampling strategy, and adjusted for clustering.

## Results

We present the characteristics of our sample in Table 1. Two-thirds of the women were between the ages of 20 and 30 (66.0%) and had never attended any formal schooling (65.3%). More than 8 in 10 were married (82.8%) and living in a rural area (81.4%). Additionally, 79.5% of women had at least one child. Only 12.2% of women were using any form of contraception, including 2.2% who were using a long-acting reversible contraceptive method. Women who reported having at least one close friend were younger and more likely to reside in a rural area than women with no friends, with no other statistically significant differences, including in relation to abortion incidence (Table 1).

**Table 1:** Characteristics of female respondents aged 15 to 49 overall and by whether report having any close female friends*.

	All respondents		0 Friends		≥ 1 Friend	
	%	N	%	N	%	N
Age						
15–19	19.6	821	**19.9**	204	**19.5**	617
20–29	40.4	1400	**33.7**	297	**42.0**	1103
30–39	25.6	932	**26.4**	249	**25.4**	683
40–49	14.5	543	**20.0**	173	**13.1**	370
Education						
Never	65.3	1747	68.1	422	64.6	1325
Primary	18.2	697	14.6	170	19.0	527
Secondary or higher	16.6	1251	17.3	331	16.4	920
Currently married						
No	17.2	1040	19.4	289	16.6	751
Yes	82.8	2656	80.6	634	83.4	2022
Wealth tertile						
Poorest	32.0	686	30.6	118	32.4	568
Middle wealth	31.9	670	29.0	126	32.6	544
Wealthiest	36.1	2340	40.3	679	35.1	1661
Residence						
Rural	81.4	1677	**75.7**	310	**82.9**	1367
Urban	18.6	2019	**24.3**	613	**17.1**	1406
Has any children						
No	20.5	1055	20.0	265	20.6	790
Yes	79.5	2638	80.0	657	79.4	1981
Currently using contraception						
No	87.8	3115	89.5	771	87.4	2344
Yes	12.2	581	10.5	152	12.6	429
Currently using LARC						
No	97.8	3578	98.0	893	97.7	2685
Yes	2.2	118	2.0	30	2.3	88
Total	100.0	3696	100.0	923	100.0	2773

*Estimates weighted, Ns unweighted; bold indicates p-value for design-based F test less than 0.05.

There were several differences between the friends and the respondents, even after adjustments to the friend data (Table 2). The adjusted friend sample age distribution was slightly older than the respondent sample. Furthermore, the adjusted friend sample had lower levels of education than the respondent sample, and friends were more likely to live in urban settings than respondents. Finally, friends were more likely to be using contraception than respondents, while current marital status, parity, and use of long-acting reversible contraception were similar in the respondent and adjusted friend samples.

**Table 2:** Characteristics of female respondents aged 15 to 49 and their closest female friends aged 15 to 49*.

	Respondent		Unadjusted friend		Adjusted friend**	
	%	N	%	N	%	N
Age						
15–19	19.6	821	19.1	556	**19.6**	786
20–29	40.4	1400	39.4	976	**38.9**	1325
30–39	25.6	932	27.1	672	**26.8**	974
40–49	14.5	543	14.4	408	**14.7**	611
Education						
Never	65.3	1747	**69.2**	1399	**67.7**	1837
Primary	18.2	697	**15.5**	462	**16.3**	641
Secondary	16.6	1251	**15.2**	884	**16.0**	1218
Currently married						
No	17.2	1040	18.8	759	18.9	1048
Yes	82.8	2656	81.2	2014	81.1	2648
Wealth tertile						705
Poorest	32.0	686	—	—	—	—
Middle wealth	31.9	670	—	—	—	—
Wealthiest	36.1	2340	—	—	—	—
Residence						
Rural	81.4	1677	**78.0**	1332	**77.9**	1642
Urban	18.6	2019	**22.0**	1440	**22.1**	2054
Has any children						
No	20.5	1055	21.9	828	21.0	1093
Yes	79.5	2638	78.1	1939	79.0	2602
Currently using contraception						
No	87.8	3115	**85.3**	2290	**85.9**	3061
Yes	12.2	581	**14.7**	483	**14.1**	635
Currently using LARC						
No	97.8	3578	97.5	2675	97.5	3568
Yes	2.2	118	2.5	98	2.5	128
Total	100.0	3696	100.0	2773	100.0	3696

*Estimates weighted, Ns unweighted; bold indicates p-value for design-based F test (reference respondents) less than 0.05.

**Estimates include respondent characteristics in place of “missing” confidantes; post-stratification weights applied.

Table 3 shows the one-year abortion incidence per 1,000 women aged 15–49 for both the respondent and the adjusted friend samples. The adjusted friend estimate was significantly higher than the respondent estimate based on the non-overlapping confidence intervals, with 6.7 abortions per 1,000 women in 2021 (95% CI: 1.3, 12.1) for friends compared to 0.4 abortions per 1,000 women (95% CI: 0.0, 0.7) for respondents. There were few significant socioeconomic differences between respondent and friend estimates with the exception of women aged 20–29 in the friend group who were significantly more likely to have had an abortion, with 4.5 abortions per 1,000 women (95% CI: 2.2, 6.7) compared to only 0.5 (95% CI 0.2, 1.7) for respondents. However, we identified no abortions among respondents who were not married or who lived in rural areas, while the friend estimate was 6.8 abortions per 1,000 women for both groups (unmarried 95% CI: 1.2, 12.3; rural 95% CI: 0.0, 13.6).

**Table 3:** Induced abortion incidence (per 1,000) among female respondents aged 15 to 49 and their closest female friends aged 15 to 49 by background characteristics.

	Respondent			Adjusted friend**		
	Rate	95% CI		Rate	95% CI	
Age						
15–19	0.6	0.1	4.4	11.5	0.0	28.6
20–29	0.5	0.2	1.7	**4.5**	**2.2**	**6.7**
30–39	0.3	0.0	2.3	9.9	0.0	24.7
40–49	0.6	0.1	4.4	0.1	0.0	0.3
Education						
Never	0.1	0.0	0.2	6.1	0.0	12.3
Primary	0.4	0.0	1.3	2.4	0.0	5.6
Secondary or higher	0.0	0.0	0.0	13.9	0.0	35.2
Currently married						
No	0.0	0.0	0.0	6.8	1.2	12.3
Yes	0.5	0.1	0.9	6.7	0.2	13.2
Wealth tertile						
Poorest	0.0	0.0	0.0	—	—	—
Middle wealth	0.0	0.0	0.0	—	—	—
Wealthiest	1.1	0.1	2.0	—	—	—
Residence						
Rural	0.0	–	–	6.8	0.0	13.6
Urban	2.1	0.1	4.0	6.5	1.2	11.8
Has any children						
No	0.5	0.0	1.5	11.3	0.0	27.5
Yes	0.3	0.0	0.6	5.5	0.2	10.8
Total	0.4	0.0	0.7	**6.7**	**1.3**	**12.1**

*Estimates weighted, Ns unweighted; bold indicates p-value for design-based F test (reference respondents) less than 0.05.

**Estimates include respondent characteristics in place of “missing” friends; post-stratification weights applied.

Respondent and friend estimates revealed somewhat different abortion characteristics (Table 4). A larger percentage of friends versus respondents used multiple methods (45.4% and 16.7%, respectively), however the difference was not significant due to the small number of abortions in each sample. Among respondents, pills other than medication abortion pills were the most common method used, with 35.9% using unidentified pills and 24.8% using other identified pills. Very few reported using a recommended method, with only 2.7% reporting having used an abortion surgery and 3.0% having used medication abortion pills. Among friends, 56.4% used a traditional or “other” method, which was significantly higher than the 2.7% of respondents who used these methods. Identified pills other than medication abortion pills (44.6%) was the next most common method used by friends. Friend use of pills that the respondent could not identify (6.8%) was significantly lower than that of respondents. No friends had a surgery but 8.4% used medication abortion pills.

**Table 4:** Details of most recent reported abortion among female respondents aged 15 to 49 and their closest female friends aged 15 to 49*.

	Respondent		Adjusted friend**		p-value
	%	N	%	N	
Used multiple methods	16.7	4	45.4	6	0.0860
All methods used***					
Surgery	2.7	1	0.0	0	0.155
Mifepristone/misoprostol pills	3.0	1	8.4	4	0.346
Other pills (identified)	24.8	5	44.6	8	0.189
Unknown pill type	35.9	6	**6.8**	3	**0.022**
Traditional/other methods	2.7	1	**56.4**	**9**	**0.000**
Do not know/No response	8.5	2	10.7	6	0.712
All sources used***					
Public facility	38.4	7	46.4	9	0.663
Private facility	6.9	1	22.7	3	0.290
Pharmacy	31.2	7	17.0	8	0.400
Other non-clinical	33.1	7	39.6	9	0.835
Do not know/No response	0.0	0	3.6	2	–

*Estimates weighted, Ns unweighted; bold indicates p-value for design-based F test (reference respondents) less than 0.05.

**Estimates include respondent characteristics in place of “missing” friends; post-stratification weights applied.

***Respondents could select multiple methods/sources thus percentages do not sum to 100%.

Both respondents and friends were most likely to seek abortion services at public facilities (38.4% and 46.4%, respectively), followed by other non-clinical sources (33.1% among respondents, 39.6% among friends) (Table 4). The third most common source of abortion services for respondents were pharmacies (31.2%) and private facilities for friends (22.7%). There were no significant differences in the types of sources used for abortions among respondent and friend samples.

We present the safety of the most recent reported abortion among female respondents and their closest friends in Table 5. There were no significant differences in the distribution of abortions involving recommended methods and/or sources between respondent and friends. Approximately half of respondents (51.7%) and friends (49.0%) had abortions involving non-recommended methods and sources, with similar proportions of abortions involving either non-recommended methods or sources for respondents (45.6%) and friends (51.0%). Very few abortions among respondents involved recommended methods and sources (2.7%) and there were none among friends. Using the updated safety measure, which recategorizes medication abortion pills obtained from outside a facility as involving a recommended method and source, the percentage of abortions in this group increased to 5.7% among respondents and 8.4% among friends.

**Table 5:** Safety of most recent reported abortion among female respondents aged 15 to 49 and their closest female friends aged 15 to 49*.

	Respondent		Adjusted friend**	
	%	N	%	N
Adapted from WHO safety measure***				
Recommended method and source	2.7	1	0.0	0
Non-recommended method or source	45.6	8	51.0	11
Non-recommended method and source	51.7	11	49.0	14
Reclassifying medication abortion pills from outside facilities as recommended source****				
Recommended method and source	5.7	2	8.4	4
Non-recommended method or source	42.6	7	42.6	9
Non-recommended method and source	51.7	11	49.0	14

*Bolding indicates statistically significantly different at the p<0.05 level (reference respondent).

**Adjusted friend data includes respondent abortion details for respondents who reported having no friends.

***Surgery and medication abortion pills from clinical source = recommended method and source (adapted from Ganatra et al. 2017) [11].

****Surgery from facility and medication abortion pills from any source = recommended method and source.

Overall, even when using the less conservative abortion safety measure, which categorizes medication abortions occurring outside of facilities as safe, unsafe abortion among respondents was high across all socio-demographic groups (Table 6). However, there were significantly higher levels of unsafe abortions among married friends (100.0%) compared to unmarried friends (59.3%) and among friends living in rural versus urban areas (100.0% versus 77.0%). There was also a borderline significant difference by parity, with higher levels of unsafe abortion among friends with any children (97.7%) compared to nulliparous friends (76.1%). Patterns were similar for respondents but did not rise to the level of statistical significance.

**Table 6:** Percent of abortions involving non-recommended method and/or source among female respondents aged 15 to 49 by background characteristics*.

	Respondent		Adjusted friend**	
	%	N	%	N
Age				
15–19	–	0	83.2	1
20–29	95.2	9	91.7	10
30–39	100.0	7	95.0	8
40–49	64.1	2	100.0	2
Education				
Never	100.0	4	94.0	5
Primary	100.0	3	100.0	5
Secondary or higher	91.0	11	83.0	11
Currently married				
No	85.0	4	**59.3**	**7**
Yes	96.3	14	**100.0**	**14**
Wealth tertile				
Poorest	100.0	1	–	–
Middle wealth	–	0	–	–
Wealthiest	93.8	17	–	–
Residence				
Rural	–	0	**100.0**	**6**
Urban	94.3	18	**77.0**	**15**
Has any children				
No	88.9	3	76.1	5
Yes	95.9	14	97.7	16
Total	94.3	18	91.6	21

*Estimates weighted, Ns unweighted; bold indicates p-value for design-based F test (reference respondents) less than 0.05.

**Estimates include respondent characteristics in place of “missing” friends; post-stratification weights applied.

## Discussion

This study estimates there were at least 7 abortions per 1,000 women of reproductive age annually in Niger in 2021, equivalent to approximately 36,800 abortions. More than 9 out 10 of these abortions were unsafe, including approximately 50% of abortions that involved non-recommended methods and sources. While the best friend method produced higher estimates of abortion incidence than direct reports (0.4 abortions per 1,000), we believe our friend results may still be an underrepresentation of the extent of abortion in the country. The best friend method performed somewhat less well in Niger than in other geographies as evidenced by the larger potential selection bias in the friend population, for whom several characteristics remained significantly different from respondents even after adjustment, and the more imprecise (and larger) transmission bias estimates as a result of low sharing of abortion experiences with friends [21,27].

Given the dearth of abortion-related data from Niger prior to this study and lack of an external objective measure, we have little country-specific information on this phenomenon to refer to in contextualizing or validating these findings. The one existing national estimate of abortion incidence in Niger is derived from models suggesting an annual rate of 15 per 1,000 in 2015–2019, which is double our adjusted friend estimate [2]. However, the model relies solely on proxy determinants of abortion incidence (percent married, fertility rates, percent with contraceptive unmet need, percent of pregnancies unintended) rather than direct measures of abortion for Niger and provides no insight on the characteristics of women who have the abortions or the safety of these procedures [8]. Regional estimates indicate 85% for abortions are unsafe, on par with our safety results [11].

Studies of abortion in neighboring countries provide additional insight into the likely extent and patterns of abortion in Niger. In Burkina Faso, which shares Niger’s southwest border, researchers estimated an abortion incidence of 23 abortions per 1,000 women aged 15–49 in 2021, more than 90% of which were considered unsafe [21]. In Kano State of Nigeria, which also borders Niger and is more religiously similar to Niger than Burkina Faso, there were an estimated 5.4 abortion per 1,000 women in 2017, however, this estimate was based on self-reported data and thus underestimates the extent of abortion in the region [22]. A non-representative survey implemented in Chad, which borders Niger to the East, estimated that 3.4% of pregnancies ended in abortion, which is much lower than the regional estimate of 12% for Western Africa, and lower even than the modeled Niger estimate of 5% [34–36]. However, Niger remains contextually distinct from its surrounding countries as it has the highest fertility and desired fertility in the world [7], the lowest rate of unintended pregnancy [8], and among the lowest levels of contraceptive use [9], thus we would anticipate lower levels of abortion than those observed in nearby countries given a lower demand for fertility regulation. In terms of abortion patterns, findings from Burkina Faso, Nigeria, and Chad suggest incidence is often higher among younger women (women in their 20s in Nigeria and Chad, teens and 20s in Burkina Faso), unmarried women, nulliparous women, those with higher levels of education, and those who lived in urban settings [21,22,34]. In Niger, when examining the adjusted friend data, we found similar patterns by age, education and parity but not by marital status or area of residence. However, our results should be interpreted with caution given the small number of abortions reported.

Almost all abortions (97% to 100%, depending on respondent versus friends) were unsafe, including half that did not involve a recommended method or recommended source. Similar results were observed in nearby Burkina Faso, with 92% of abortions considered unsafe [21]. While our abortion numbers were small, unsafe abortions seemed more common among older women, married women, those with children, and those residing in rural areas based on the friend data. Research from other sub-Saharan African settings reveals similar patterns of disparities in abortion safety [22,23,37–39].

There are several important limitations to consider in interpreting these results. A central assumption of social network-based methods is that friends represent a surrogate sample that is similar to respondents in observed and unobserved ways. However, roughly 1 in 4 women in Niger reported having no close female friends, introducing potential bias in the surrogate sample. The adjusted friend sample was still significantly different than the respondent sample after adjustment, although the largest difference (for residence) only reached 3.5 percentage points. The method also assumes that respondents know about their closest friend’s abortion experiences. However, we observed very low abortion reporting among respondents (n = 20), 13 of which had a close female friend, and four of whom (29%) shared their abortion with that friend. This resulted in a transmission bias adjustment factor of 3.45. In other settings the level of sharing was 50% or higher, resulting in adjustment factors around 2.0 or less [21,24,26,40], indicating the stigma surrounding abortion may be much higher in Niger. This adjustment for transmission bias [24–26] also assumes abortion sharing patterns are similar in both directions (respondents to friends and friends to respondents) and that sharing among those who reported their abortion in the survey accurately reflects sharing among those who did not report their abortion on the survey, which we cannot validate. Relatedly, respondents reported on relatively few friend abortions (n = 26), likely due to this visibility bias. Additionally, the inclusion of reports of menstrual regulation may capture non-abortions as women may take action to bring back their period for a number of reasons [30,31], however, we sought to minimize this risk by specifying instances where the woman thought she was pregnant and was successful in bringing back her period. Lastly, our assessment of abortion safety is limited by the details we could reliably collect from respondents about their and their friends’ abortions. We may have overestimated the extent of safe abortions given the lack of information on provider training for surgical abortions and medication abortion dosage and timing. Information on use of appropriate methods (and from clinical sources in the case of surgeries), does not inherently mean the abortion was performed in accordance with recommendations [38,41–46], nor does it capture other aspects of safety and quality of care that are important in considering the abortion experience [47,48].

Despite these limitations, this is a first attempt to study abortion incidence and safety at a national level in Niger. We used a large, population-based dataset to collect information on abortion experiences, addressing an important gap in our understanding of this reproductive event in Niger. Our findings illustrate that, despite legal restrictions and substantial stigma, some women undergo abortions to manage their fertility at great risk to their physical and social safety. Nearly all abortions are unsafe, involving non-recommended methods and/or sources.

Ensuring adequate access to quality voluntary family planning services to prevent unintended pregnancy is essential to reducing the risk of unsafe abortion and its negative sequelae in Niger. In the absence of legal reform, postabortion care will continue to be an essential aspect of emergency obstetric care needed to reduce the burden of unsafe abortion-related morbidity and mortality in this country. As in other settings, unregulated diffusion of safe medication abortion pills is also likely to mitigate abortion complications even without legal reform.
